# Understanding the reactivity of copper current collectors in lithium batteries – an *operando* XAS study

**DOI:** 10.1039/d6eb00072j

**Published:** 2026-08-24

**Authors:** M. A. Koronfel, A. Omirkhan, H. Yadegari, D. Thornton, W. Xue, J. S. Zhang, L. Guo, J. Nelson Weker, J. F. W. Mosselemans, M. Kulzick, I. E. L. Stephens, M. P. Ryan

**Affiliations:** a Imperial College London, Department of Materials and London Centre for Nanotechnology London SW7 2AZ UK aigerim.omirkhan@imperial.ac.uk; b The Faraday Institution, Quad One Harwell Science and Innovation Campus Didcot OX11 0RA UK; c SLAC National Accelerator Laboratory Menlo Park 94025-7015 USA; d Diamond Light Source, Rutherford Appleton Laboratory Didcot OX11 0DE UK; e BP Corporate Research Center Naperville Illinois 60563 USA

## Abstract

Copper is used as the anode current collector in lithium-ion batteries, as well as the electrode itself in other types of lithium batteries. It is nominally a stable material in the organic electrolytes under normal battery operating potentials. Copper dissolution in lithium battery electrolytes has been extensively shown to occur at potentials attainable only in over-discharge conditions. However, in this work, copper dissolution is shown to occur even under normal cycling conditions, contrary to thermodynamic prediction. Utilising the high chemical sensitivity of X-ray absorption spectroscopy, copper oxidation state and concentration are quantified *in operando*. The results raise concerns about the impact of copper on battery efficiency, lifetime and safety, along with resulting implications on battery cycling.

## Abstract 2

Tracking oxidation of copper current collector in *operando*.

## Graphical abstract

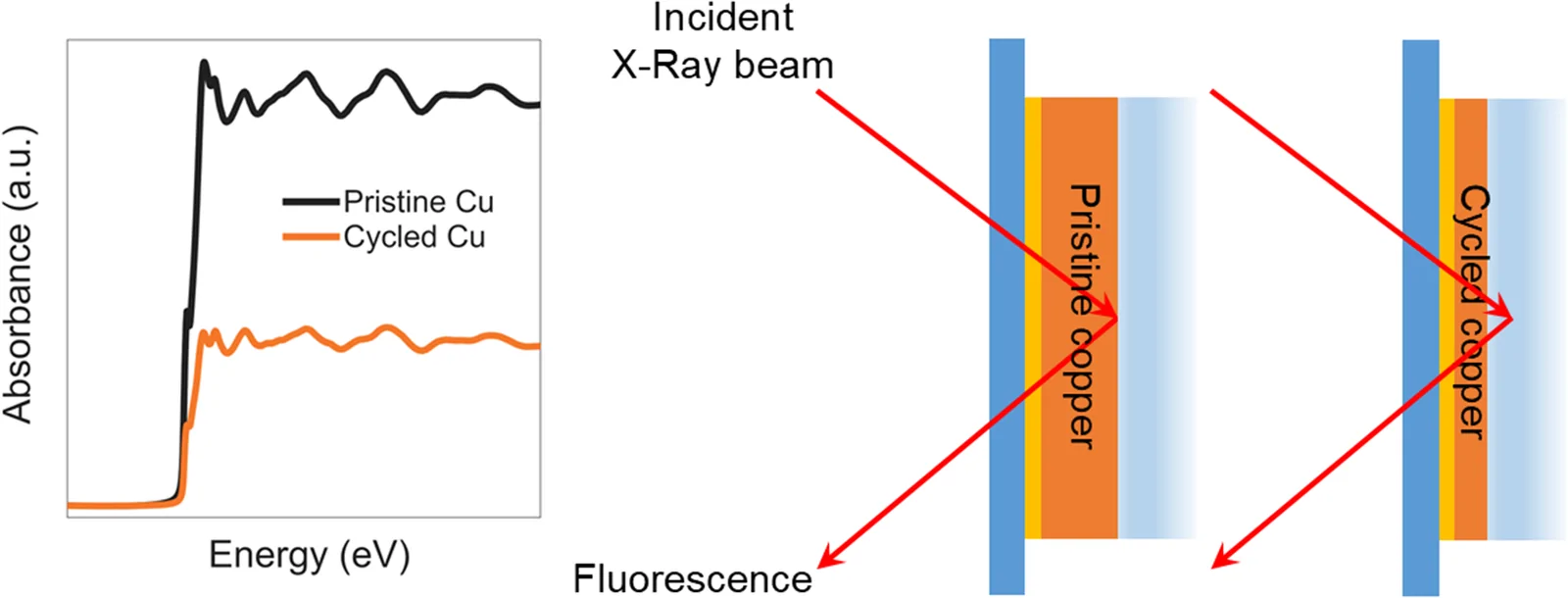

**Broader context**

Copper plays a critical role in lithium-ion batteries, serving both as a current collector and, increasingly, as the active anode in emerging anode-free cell designs. Under normal operating voltage windows of the battery, copper is thought to be thermodynamically protected from corrosion in lithium-ion batteries. Here, we challenge this assumption by demonstrating that copper dissolution and re-deposition occur even under routine conditions during open circuit rest and cycling of the batteries. *Operando* X-ray absorption spectroscopy (XAS), which tracks copper speciation, shows that dissolved copper species participate in redox reactions during battery cycling, ultimately re-depositing on electrodes. We provide mechanistic explanation for this phenomenon whereby the oxidation potential of copper is lowered due to the presence of trace moisture and oxygen along with the *in situ*-generated oxidative species that arise from ongoing electrolyte and interface degradation. These insights have broad implications for energy storage research: they highlight the importance of stabilising anode environments and designing battery management strategies such as tap-charging that suppress the chemical conditions enabling copper dissolution. More broadly, this work exemplifies how advanced *operando* spectroscopy can resolve hidden degradation pathways, guiding the development of more durable and safer batteries.

## Introduction

Lithium-ion batteries (LIBs) currently offer the best energy storage performance for electric vehicles. Since their first commercialisation in 1991,^1^ the energy density of LIBs has grown by almost threefold (from 90 Wh kg^−1^ to 250 Wh kg^−1^). Despite the widespread uptake of LIBs in state-of-the-art electric vehicles, issues with battery degradation reducing both lifetime and safety persist.

LIBs operate through the migration of Li ions and electrons from the positive electrode (cathode) to the negative electrode (anode) during charge and *vice versa* during discharge. Whilst the Li ions flow through the electrolyte and a permeable separator between the two electrodes, electrons are collected by current collectors and flow through the external load to deliver power. Current LIBs employ a transition metal oxide or phosphate cathode (*e.g.* LiCoO_2_, LiNiMnCoO_2_, LiFePO_4_) coupled with a graphite anode and a lithium-salt based organic liquid electrolyte. The current collectors are aluminium and copper foils at the cathode and anode electrodes respectively. Although research continues for higher capacity and less expensive battery chemistries, extensive work is also ongoing on mitigating the degradation mechanisms of existing battery technology. Multiple degradation mechanisms lead to discrepancies between theoretical and practical capacities of LIBs, capacity fade, as well as potential safety hazards. The degradation processes^2^ include: electrolyte decomposition (leading to the formation of the solid electrolyte interphase (SEI)),^3^ binder detachment,^4^ solvent co-intercalation,^5^ active material dissolution,^6^ gas evolution,^7,8^ lithium plating^9,10^ and current collector dissolution.^11^ The mechanisms arising at the electrode–electrolyte interface have understandably gained the most attention (since these interactions and materials are what largely determine the battery capacity). However, issues at the current collector–electrode interface can also significantly affect the efficiency of LIBs. Dissolution of current collectors would lead to the production of corrosion products that could reduce cell efficiencies by increasing electronic resistance,^12^ as well as limiting intercalation and de-intercalation of lithium ions.^13,14^

This work focuses on elucidating the reactivity of copper, within normal cell operating potentials, through a highly sensitive and quantitative approach. The copper redox is studied in three different types of lithium cell: (i) full lithium-ion cell assemblies (Cu/graphite anode *versus* transition metal oxide/Al cathode), (ii) anode-free cells (copper *versus* transition metal oxide cathode), and (iii) copper *versus* lithium *quasi-symmetrical* cells. Anode-free cells (AFC), also known as anode-less lithium batteries,††We use “anode-free cell” (AFC) only as a common shorthand. In practice, no cell is truly anode-free once lithium plates during the first charge. As discussed by Hatzell,^41^ these systems are more accurately described as Li-free or low N/P lithium-metal batteries. contain no active lithium-ion host at the anode and have attracted recent research interest.^15–18^ In AFCs lithium ions from the cathode are plated directly on the copper current collector during charge and stripped back to the cathode during discharge. In this work, the AFCs and the *quasi-symmetrical* (Cu∥Li) cells were utilised to elucidate the effect of removing the graphite coating on the behaviour of copper current collectors.

Based on previous thermodynamic calculations, copper dissolution in LIBs is only expected under *over-discharge* conditions. The open circuit potential (OCP) of copper in LiPF_6_ electrolytes is approximately 3–3.3 V (*vs.* Li/Li^+^, against which all potentials in this work are reported).^19,20^ The onset of copper oxidation in LIB electrolytes has generally been reported to occur at potentials above 3.5 V.^19–23^ He *et al.*^22^ reported the oxidation potentials for copper to Cu(i) and Cu(ii) at 3.92 V and 4.17 V respectively using cyclic voltammetry. During normal operating conditions, the potential of the anode is well below the OCP of copper in the electrolyte (usually < 1 V) and hence copper is expected to be immune to dissolution. In LIB assemblies, some cells may be susceptible to over-discharge due to the capacity and voltage variation (*i.e.* nominal *vs.* actual voltage seen by individual cell) from heterogeneity across the cells in the pack. The cell with the lowest capacity may experience over-charge and over-discharge even when the surrounding cells are experiencing normal charge and discharge conditions. During over-discharge the potential of the anode increases dramatically, and potentially beyond the onset of copper oxidation. Several reports of both modelling and experimental studies have shown the oxidation of copper in over-discharged cells and its subsequent distribution throughout the cell and re-deposition as metallic copper on the anode, cathode and separator.^14,21,22,24–29^ Nonetheless, copper dissolution has been reported after just immersion tests in electrolyte at OCP (up to 720 h) and was hypothesised to be due to the presence of trace H_2_O impurities.^30,31^ Despite this detection of copper dissolution at OCP, copper dissolution in lithium-ion systems is generally neglected.

In this work, methodologies to monitor the oxidation state of copper during normal cycling conditions of cells have been developed. The high chemical resolution of synchrotron-based X-ray absorption spectroscopy (XAS) was employed accompanied with a setup that allowed the monitoring of extremely small changes in the oxidation state and concentration of copper. XAS is a high-brightness, element-specific technique that probes the local electronic and atomic structure of materials by measuring how X-rays are absorbed as a function of energy near an element's absorption edge. When performed at synchrotron sources, XAS offers high energy resolution and time-resolved capabilities, making it ideal for *operando* studies of battery electrodes under realistic cycling conditions. The copper was studied as 20 nm thin films and Cu K-edge XAS spectra were collected *in operando*. The cell designs and fluorescence geometry XAS were set so that measurements were obtained at the Cu/electrode (or Cu/electrolyte) interface. The penetration depth of X-rays meant that the full thickness of the Cu films would be sampled by the X-rays and any changes to the amount or speciation of the full film depth would be recorded. The use of thin films both facilitated this approach and meant that surface changes were a significant fraction of the total signal, allowing for enhanced sensitivity for dissolution measurements. See SI Fig. S3 for a schematic illustrating key XAS spectral features: edge, edge-height, along with short introduction to XAS.

## Experimental

### Materials and cell construction

Copper thin films were grown on 8 µm Kapton foils (Advent Research Materials, UK), to a thickness of 20 nm, by sputtering using LAB 18 thin film deposition system (Kurt J. Lesker) and a copper sputtering target (Testbourne Ltd, UK).

Three types of lithium cells were studied: full lithium-ion cells (LIC), anode-free cells (AFC), and Cu∥Li cells *quasi-symmetrical* cells (QSC). The copper thin film was directly utilised as the positive electrode against lithium foil (Merck, UK) in QSC, as well as the negative electrode against a polycrystalline (PC) LiNi_0.8_Mn_0.1_Co_0.1_O_2_ (NMC811, Targray, Canada) positive electrode in AFC. Additionally, the copper thin film was the current collector of a natural graphite (Elcora Advanced Materials, Canada) negative electrode against NMC811 positive electrode in LIC.

NMC811 and graphite electrodes were prepared as slurries in an anhydrous *N*-methyl-2-pyrrolidone (Sigma Aldrich, UK) at 90 : 5 : 5 wt% (NMC : carbon black : polyvinylidene fluoride (PVDF) binder) and 95 : 5 wt% (graphite : PVDF). NMC was tape cast on an aluminium foil current collector and dried in vacuum at 80 °C overnight. Graphite was tape cast on the copper coated Kapton foils and dried in ultra-high vacuum overnight at room temperature, to avoid oxidising the thin copper film. 316L stainless steel foil (Advent Research Materials, UK) was used as the current collector for Li electrodes.

In NMC811 containing cells (AFC and LIC), three glass-fibre separators‡‡Three separators were used in the NMC containing cells to increase the X-ray path and reduce fluorescence signals from the cathode (Ni or any plated Cu on the NMC). This improves the signal to noise ratio of the copper oxidation state measurement at the anode current collector and ensures the measurement is limited to the current collector/electrolyte interface. (Whatman) were used, while in QSC, a Celgard separator was used. The separators were pre-soaked in an electrolyte of 1 M lithium hexafluorophosphate (LiFP_6_) solution in ethylene carbonate (EC) and ethyl methyl carbonate (EMC) (3 : 7 weight ratio).

All cells were built inside an argon-filled glovebox, and two types of cell housing were employed: customised coin cells CR2023 and X-ray transparent battery pouch cells (SI Fig. S1). The coin-cells were initially used for LIC and AFC. They comprised a 7 mm diameter hole in one of the caps on which the Kapton backed electrode was glued using epoxy glue. QSC were built in laminated aluminium-based pouches (Sorbent Systems, USA), which are heat sealed and relatively X-ray transparent, hence require no specific X-ray window (copper-coated Kapton sheets were still used as the electrodes *inside* the pouch). The pouch cells were introduced for better insulation from outer atmosphere as they offer significantly lower oxygen permeability compared to the Kapton window employed in coin cells.

### X-ray absorption spectroscopy (XAS)

Cu K-edge XAS was carried out at beamlines I20 of Diamond Light Source (DLS), UK and 10-BM of the Advanced Photon Source (APS), USA. Studies on LIC and AFC were conducted at DLS where the beam size (sampled area) was 1 mm × 1 mm, while experiments on QSC were carried out at APS where the beam size was 0.5 mm × 3 mm. The collection time for a single spectrum was 7 minutes at DLS I20 (for an energy range from 8890 eV to 9250 eV) and 14 minutes at 10-BM at APS (for an energy range from 8890 eV to 9050 eV).

The cells were set up so that the Kapton/Cu thin film formed the X-ray window. The XAS scans were collected in fluorescence geometry whereby the cells and the detector were mounted at 45° and 90° to the incident beam respectively (SI Fig. 2). This geometry was specifically selected to probe the Cu thin film and the immediate Cu/electrolyte interfacial region. Consequently, the intensity of the fluorescence signal is directly proportional to the amount of Cu remaining, within the probed region, on the Kapton substrate. As Cu species dissolve and diffuse away from the illuminated interfacial region into the bulk electrolyte, they no longer contribute appreciably to the measured fluorescence signal. The resulting decrease in the Cu K-edge step therefore provides a sensitive *in situ* measure of the loss of Cu from the thin film current collector.

Importantly, the experiment was not configured to quantify the total concentration of dissolved Cu^+^/Cu^2+^ species within the bulk electrolyte. Instead, the measurement was designed to simultaneously track changes in the amount of copper remaining at the current collector and changes in its chemical state, including the Cu(0), Cu(i), and Cu(ii) contributions associated with the film and its surface oxide/interphase. This distinction enables both the dissolution and chemical evolution of the copper current collector to be followed directly during electrochemical operation.

X-ray absorption spectra were recorded first at OCP followed by recording *in operando* as a function of time and potential. Changes in Cu chemical composition (oxidation states) would result in changes in the edge position and structure, while changes in local concentrations would result in changes to the edge-step height (intensity changes). To monitor the chemical changes the spectra were background-subtracted, normalised and then fit, using linear combination fitting (LCF), with standard copper spectra. These standards were copper metal foil, as well as Cu(i) and Cu(ii) oxides (10 wt% of Cu_2_O and CuO in boron nitride pellets). LCF determines the proportions of the standards’ spectra that, when summed, yield the least squares fit to the spectrum of interest. To analyse the local concentration of copper, only background-subtracted spectra are used to record the variation in edge-step intensity between the different spectra.

## Results and discussion

### Copper dissolution at OCP

**Fig. 1:**
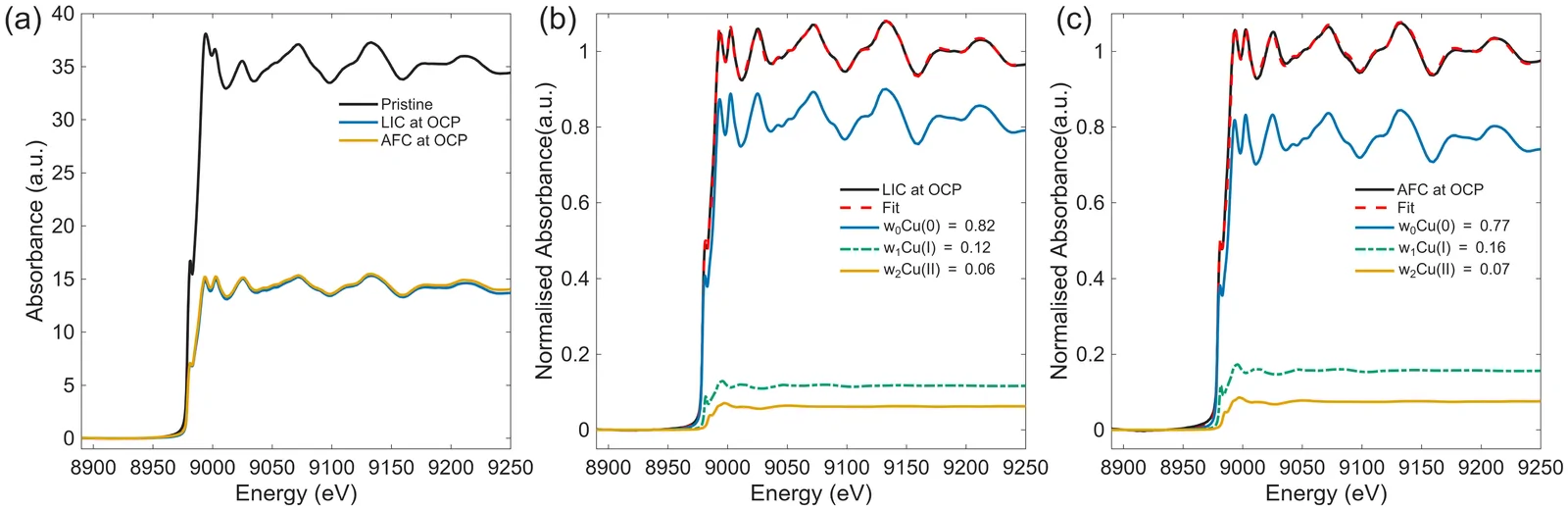
(a) Un-normalised X-ray absorption spectra showing the difference in edge-step (concentration of copper) in a pristine copper thin film, graphite-coated film assembled in a full lithium-ion cell (LIC), and an uncoated film inside an anode free cell (AFC) at Open Circuit Potential (OCP). (b and c) LCF results of the graphite-coated (b) and the uncoated (c) copper films revealing their chemical composition at OCP pre-cycling (*t* < 0 s).

Fig. 1(a) compares the (un-normalised) X-ray absorption spectrum of an as-deposited pristine copper thin film (*i.e.* unexposed to electrolyte) to those of copper in AFC and graphite-coated copper in LIC at OCP (prior to cycling) 72 h after cell assembly. In this measurement geometry, the variation in edge-step of un-normalised spectra is directly proportional to the copper concentration. The edge-step decreased from 35 a.u. for the pristine film to approximately 14 a.u. for the *in situ* films. This indicates 60% dissolution of the copper film during the 72 h period between the cells’ assembly and the time of measurement (*i.e.* open circuit and not tap charged). This corresponds to an estimated ∼12 nm dissolution of the original 20 nm film (assuming uniform dissolution). Although this is a significant rate of dissolution, in practice commercial cells are likely to have significantly less H_2_O and O_2_ contamination compared to the customised coin cells used in this study. Additionally, as the dissolution rate will decline over time in a closed system, this single time-point measurement does not reflect the long-term behaviour of commercial cells. Nonetheless, potential copper dissolution at OCP presents a significant challenge for improving the lifetime of lithium-ion batteries as corrosion species are likely to affect the electronic resistance of the cell as well as (de)intercalation of Li^+^ ions. The measured ∼12 nm loss corresponds, on a purely geometric basis, to ∼0.2% of a typical 7–8 µm thick Cu foil used in commercial cells. This estimate should be regarded as an order-of-magnitude indication rather than a direct quantitative extrapolation since dissolution kinetics may depend on surface and microstructural state.

The composition of the films at OCP, prior to cycling (*t* < 0 s), was calculated through fitting the measured spectra *χ* with the linear combination of the Cu standards (*χ* = *w*_0_·Cu(0) + *w*_1_·Cu(i) + *w*_2_·Cu(ii)) where composition fractions of Cu(0), Cu(i), Cu(ii): *w*_0_, *w*_1_ and *w*_2_ are optimised to achieve the least squares fit. The goodness of fit was assessed using the *R*-factor, which quantifies the agreement between the experimental and fitted spectra; the corresponding distribution of *R*-factor values is presented in SI Fig. S4, and indicates the fits were robust.

The results shown in Fig. 1(b and c) reveal 18% oxide surface layer (*w*_1_ + *w*_2_) on the graphite-coated copper in LIC compared to 23% on the uncoated copper in AFC configuration. This initial finding of compositional difference between graphite-coated Cu and uncoated Cu films serves as an early indicator of possible synergistic protection provided by graphite (see further discussion in the “The graphite effect” subsection).

The OCP of the battery is the potential difference between the cathode and anode under zero-current conditions, arising from the difference in the electrochemical potential of lithium (or the charge carrier) between the two electrodes at their respective equilibrium or steady-state mixed potentials. Assuming normal operating conditions (*i.e.* no overdischarge) the potential of graphite-coated and uncoated copper anodes is highest under freshly assembled OCP conditions (∼3–3.3 V^19,20^), placing it close to the expected copper oxidation threshold (which can be as low as ∼3.1 V^32^). This explains the spontaneous copper dissolution observed at OCP in the uncharged LIC and AFC cells. To mitigate this degradation pathway Li-ion full cells are typically ‘tap charged’ to a cell potential ∼1.5 V. This initial polarisation results in the insertion of tiny amounts of lithium into graphite structure, lowering the anode potential. Consequently, the copper current collector is brought into a zone of cathodic protection to arrest dissolution during the electrolyte wetting phase.^11^ In the absence of tap charging, such as in these experiments, AFC (NMC *vs.* copper) resulted in the lowest metallic copper film composition at OCP compared to LIC and QSC.

### In *operando* redox

**Fig. 2:**
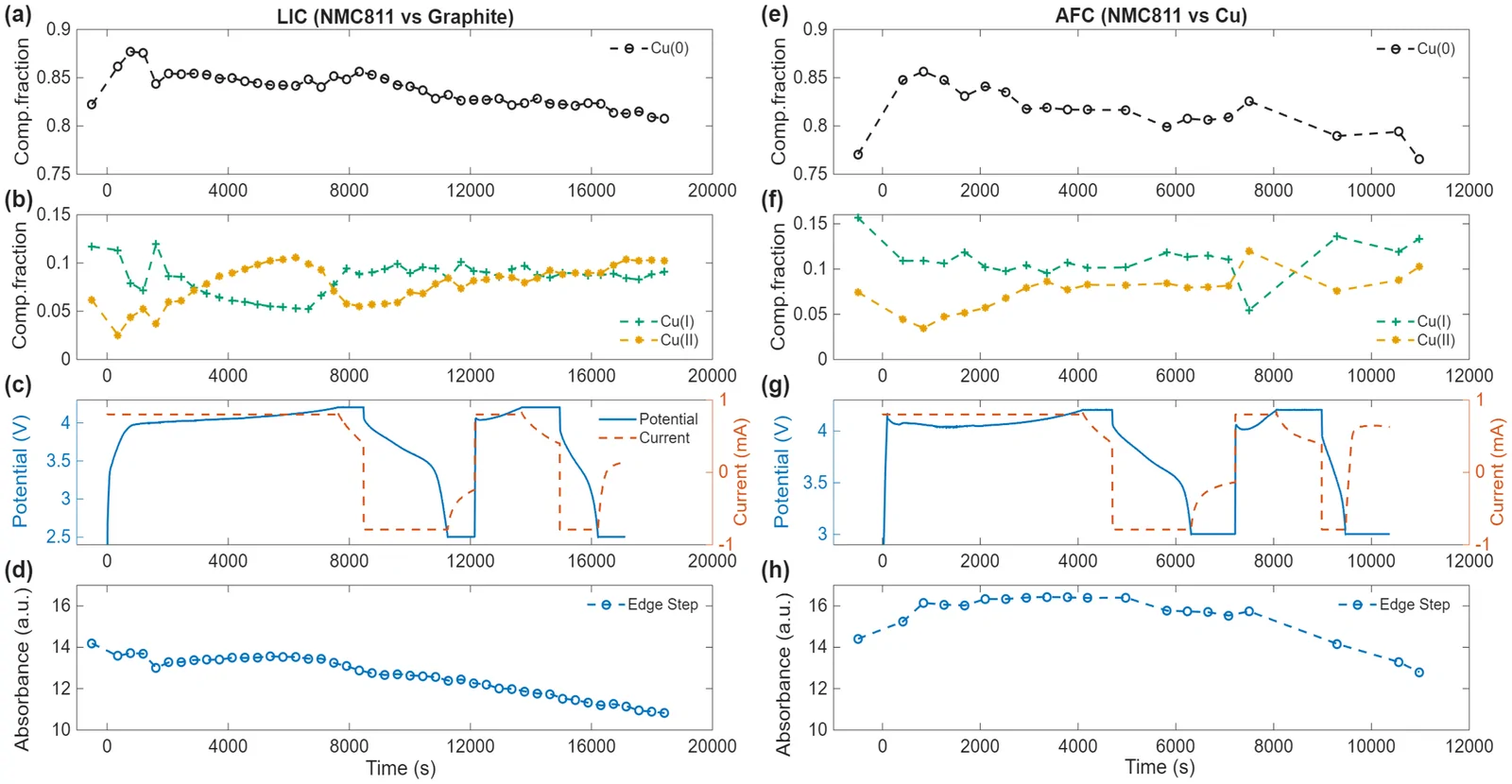
The composition fraction of the copper film as a function of time during cycling of the LIC (a, b) and AFC (e, f). (c and g) The corresponding potential and current profiles as a function of time. The cells are cycled at estimated rate of 2C using constant current – constant voltage technique between 2.5–4.2 V (LIC) and 3.0–4.2 V (AFC). Electrochemical data plotting convention: IUPAC. LIC coin cells: full cells with PC NMC811 *vs.* graphite, AFC coin cells: anode free cells with PC NMC811 *vs.* copper. The separator used was three layers of glass fibre pre-soaked in LiPF_6_ in 3 : 7 weight ratio of EC : EMC solvent. The cells are cycled at room temperature and reported as one-time measurement. (d and h) The edge step as a function of time showing changes in the overall Cu concentration during cycling.

**Fig. 3:**
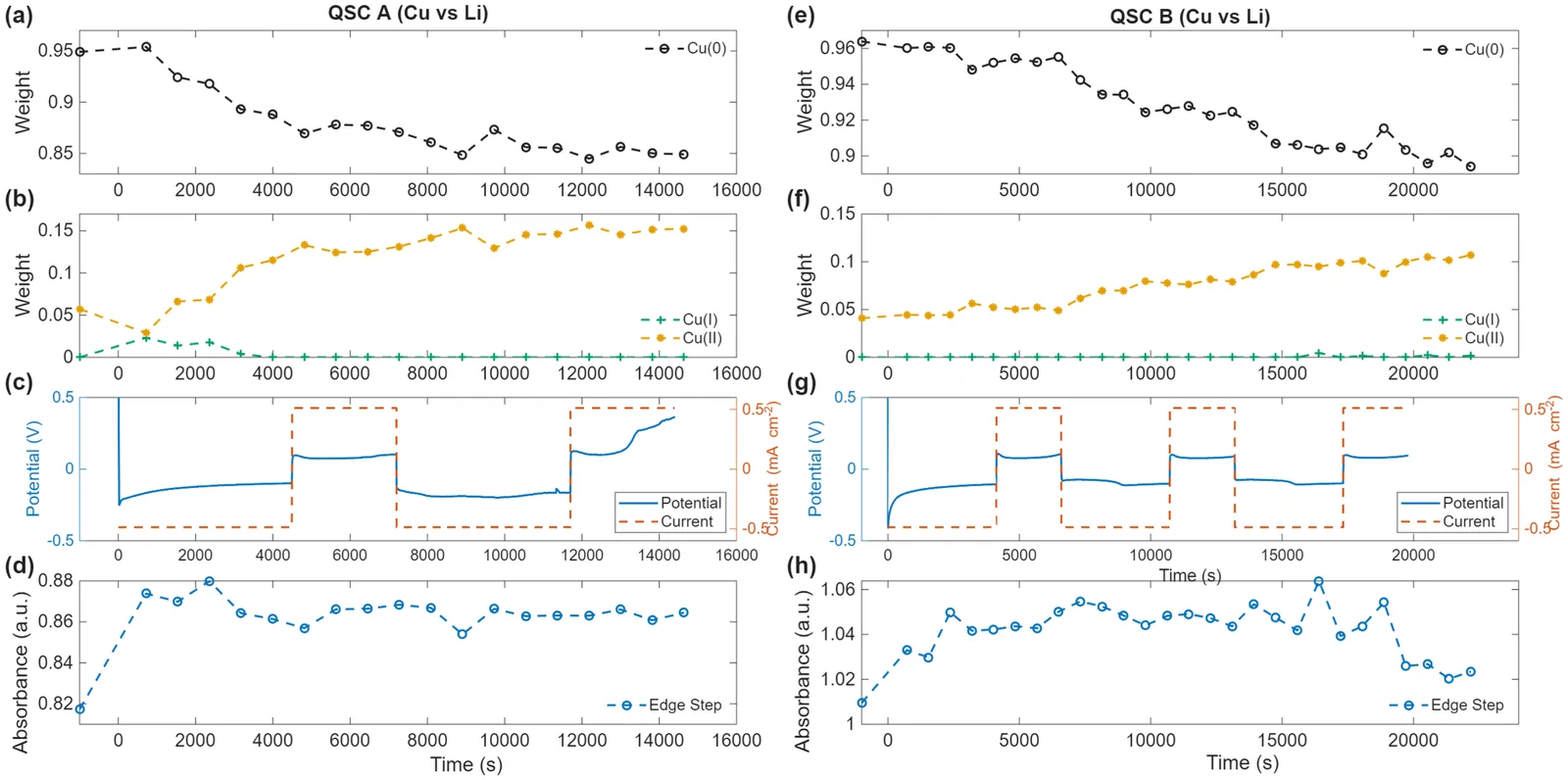
(a, b, e, f) The composition fraction of the copper film as a function of time during cycling of the two QSCs. (c and g) The corresponding potential and current profiles as a function of time. The QSCs were cycled at 0.5 mA cm^−2^ for 75 minutes charge and −0.5 mA cm^−2^ for 45 min discharge. The cells are cycled at room temperature and reported as one-time measurement. Electrochemical data plotting convention is IUPAC. QSC cells: quasi-symmetrical pouch cells with copper film *vs.* lithium foil. The separator used was Celgard pre-soaked in 1 M LiPF_6_ in EC : EMC solvent in 3 : 7 weight ratio (d and h) The edge step as a function of time showing changes in the overall Cu concentration during cycling.

Following the 72 h OCP, the LICs and AFCs were cycled using constant current constant potential technique at an estimated rates of 2C for two cycles (Fig. 2(c & g)). No initial formation cycles were conducted; hence a long initial charge is observed due to the formation of the SEI layers. The QSCs were cycled at ±0.5 mA cm^−2^ for 75 minutes charge and 45 min discharge (Fig. 3(c & g)). Here, “charge” denotes lithium plating onto copper, while “discharge” denotes stripping of the deposited lithium from the copper.

Multiple X-ray absorption spectra were measured during cycling, and LCF analysis was computed (as described in “Copper dissolution at OCP” subsection) to monitor the chemical composition of the copper films *in operando*. The LCF results at OCP pre-cycling for the LIC and AFC were presented in Fig. 1(b and c) above. For further illustration of the fitting analysis, Fig. S5 in the SI shows representative LCF fits for the LIC, during cycling, at times = 360, 9600 and 18 420 s. The composition fractions of Cu(0), Cu(i), Cu(ii) are shown as a function of time from the LIC and AFC in Fig. 2(a, b & e, f), and from two QSCs (QSC A and QSC B) in Fig. 3(a, b & e, f). The first data point in all cases (at time < 0) presents the initial composition of the films at OCP before cycling. It can be observed that the initial composition of the bare copper films in the QSC pouches was more metallic, ≥95% Cu(0) (Fig. 3(a & e)), compared to that of the AFC coin-cell, 77% Cu(0) (Fig. 2(e)). This serves as a confirmation that concerns of O_2_/H_2_O, and subsequent HF, contamination was largely alleviated in the pouch cells due to the superior sealing compared to that of the coin cells with the Kapton window.

Besides measuring the average oxidation state through the LCF analysis, the changes in the edge-steps of the Cu spectra as a function of time were also monitored to reveal changes to the local concentration of copper (*i.e.* thickness of the copper film) in all cells (LIC and AFC in Fig. 2(d & h) and QSCs in Fig. 3(d & h)).

Once cycling commenced, an initial increase in the composition fraction of Cu(0) (and drop in oxide composition fractions) was observed in almost all cells within the first measurement. This is indicative of a reduction reaction during the first charge when the potential of the anode drops rapidly from its values at OCP (∼3–3.3 V) to potentials less than 1 V. Simultaneously, the edge-step *increased* in all cells with a bare copper electrode (*i.e.* the QSCs and AFC) as soon as cycling was initiated, implying an increase in copper concentration at the current collector. These two simultaneous observations can be explained by a *re-deposition* of copper ions (which had dissolved at OCP pre-cycling) on to the anode surface as metallic copper. The re-deposition of copper would contribute to both the increase in overall copper concentration (*i.e.* the increase in the edge-step) and the increase in the metallic Cu(0) composition fraction. Contrary to AFC and QSCs, the initial increase in edge-step was not observed on the graphite-coated copper (LIC). However, this does not mean copper re-deposition does not occur in the graphite-coated copper system (as will be shown later, the re-deposition of copper on to *graphite* was confirmed by SEM-EDX as discussed in the “Copper Re-deposition on Graphite and NMC” subsection below). Instead, the dissolved copper would likely re-deposit on the graphite surface by the graphite/electrolyte interface (away from the X-ray window) and beyond the detection depth of the measurement (the copper fluorescence signal would be attenuated by the graphite coating). Although copper re-deposition on the graphite was not detected in the XAS (as evident by the absence of an increase in the edge-step), an initial reduction reaction was still observed on the LIC, as indicated by an increase in the metallic Cu(0) composition fraction as soon as cycling commenced (Fig. 2(a)). This suggests that, in addition to the reduction and plating of dissolved copper ions, another copper reduction process occurred, which is most likely the reduction of the surface oxide layer on the copper film.

As discussed earlier, copper corrosion in Li-ion cells is typically reported at potentials expected only during over-discharge, when anode potentials exceed *ca.* 3.5 V.^19–23^ The observations here of pre-dissolution of copper at OCP and its re-deposition at the onset of charging, as the anode potential decreases, suggest that the mixed potentials involving copper redox reactions have been shifted to more negative potentials compared to thermodynamic expectations. The presence of water and/or oxygen impurities could cause this negative shift. In addition to directly oxidising copper, water leads to a more acidic environment by facilitating the decomposition of the LiFP_6_ electrolyte salt to hydrofluoric acid (HF).^30^ Significant water contamination can be introduced in lithium-ion cells when the hygroscopic cathode materials are exposed to air during electrode fabrication.^33^ HF has also been shown to be generated *via* electrochemically generated proton and LiPF_6_ salt reaction within normal battery operating voltages.^34^ Last but not least, Thornton *et al.*^8^ recorded evolution of O_2_ and H_2_ in NMC *vs.* Li half-cells and evolution of H_2_ in graphite *vs.* Li half-cells. Furthermore, gases such as CO, which form as a result of solvent oxidation at the cathode, and C_2_H_4_ that forms *via* reductive decomposition of EC during SEI formation as well as CO_2_ reduction, possibly mediated by copper were detected. O_2_-derived oxidants and acids change local interfacial chemistry to favour copper dissolution within normal operating windows.

After the initial reduction of copper, a general copper oxidation trend was observed in all cells during the remainder of the cycling (Fig. 2(a & e) and 3(a & e)). Overall, the films were oxidised by 5–10% (from the most reduced state) by the end of the cycling, which spanned periods between 3 and 5.5 h. Importantly, this oxidation trend was reproducibly observed across both cell configurations, including the pouch cell QSCs, for which substantially less environmental ingress is expected than for the coin cell LIC and AFCs with a Kapton window. As discussed above, following the pre-cycling OCP period, the Cu films in the QSCs remained ≥95% metallic, compared with the 77–82% in the LIC and AFC, indicating substantially less pre-cycling oxidation in the pouch cell configuration. Nevertheless, progressive copper oxidation was observed during subsequent cycling of both QSCs, as well as the LIC and AFC. The occurrence of this trend across cells exhibiting markedly different degrees of pre-cycling oxidation indicates that the cycling-induced oxidation cannot readily be attributed solely to environmental ingress or associated O_2_/H_2_O contamination of the Kapton-window cells.

Following the early rapid decrease in anode potentials at the onset of the first charge, the anode potential is expected to remain between 0 and 1 V during normal cycling (significantly below the expected copper oxidation potential) where copper should be cathodically protected. Therefore, the oxidation of copper during normal cycling conditions was unexpected and to our knowledge previously unreported. It is noted that, apart from the initial reduction at the onset of cycling, the subsequent oxidation trend persisted irrespective of the charge/discharge direction. As copper is expected to be cathodically protected during both charge and discharge directions, significant variations in Cu(i)/Cu(ii) concentration strictly linked to current direction were not expected. The observed changes may instead involve parasitic or chemically driven processes at the Cu/electrolyte interface. A possible contribution could be chelation effects between copper and the organic molecules from the electrolyte. Complexation with organic molecules can significantly shift the oxidation potential of copper to more negative values.^35^ Furthermore, other process involving electrolyte decomposition products or local transient conditions not captured by the macroscopic potential may also contribute. These findings highlight the complexity of copper surface chemistry under realistic operating conditions and underscore the need for further mechanistic investigation.

Given the small thickness of the copper films used here (20 nm) and the significant copper dissolution at OCP (as discussed in the “Copper dissolution at OCP” subsection), progressive dissolution could potentially disrupt the electrical continuity of the film and produce isolated copper regions. Such regions would no longer be cathodically protected by the electrode potential, upon cycling, and could therefore continue to undergo reactions more characteristic of OCP conditions. However, the observed initial reduction/deposition of copper upon the onset of cycling demonstrates that the copper within the probed regions was initially electronically connected and responsive to the applied electrode potential. Electronic isolation therefore does not readily account for the onset of the observed oxidation behaviour.

The distribution of the oxidised Cu species differed between the cell configurations. Both Cu(i) and Cu(ii) contributions were resolved in the LIC and AFC, whereas the oxidised Cu contribution in the QSCs was assigned predominantly to Cu(ii). The origin of this difference cannot be established from the present measurements. However, Cu(ii) dominated speciation is not unprecedented in Li-ion battery electrolytes. Hanf *et al.*^31^ measured dissolved copper ions following exposure of copper current collectors to an LiPF_6_ in EC : EMC electrolyte under an inert atmosphere and found Cu(ii) ions comprising the majority of the dissolved ionic copper. The authors attributed this to the instability of Cu(i) ions in the electrolyte and its subsequent disproportionation to Cu(0) and Cu(ii). While such Cu(i) instability provides a possible secondary pathway contributing to the Cu(ii)-dominated speciation observed here, direct comparison between the two studies requires caution. The present XAS measurements were primarily designed to probe the Cu film and its immediate interfacial region rather than Cu species that have diffused into the bulk electrolyte. However, the relative contribution of electrolyte-phase Cu to the measured signal may have been greater in the QSC experiments. Unlike the graphite-coated electrodes used in the coin cells, the QSCs contained uncoated Cu films, eliminating attenuation by the graphite layer and potentially increasing the effective probing depth into the adjacent electrolyte. In addition, pouch cell QSCs differ from coin cells in several respects, including cell compression and potentially the extent of gas generation. These factors may have affected the effective X-ray sampling volume and, consequently, the volume of electrolyte contributing to the measured signal.

Furthermore, consistent with a greater contribution from electrolyte-phase Cu, the Cu K-edge step was comparatively stable during cycling of the pouch cell QSCs (Fig. 3(d & h)), despite the progressive change in copper oxidation state, whereas a more pronounced decrease was observed in the coin cell LIC and AFC experiments (Fig. 2(d & h)). This raises the possibility that dissolved Cu species remaining within the X-ray-sensitive volume contributed more substantially to the QSC spectra. Under such conditions, conversion of unstable dissolved Cu(i) to Cu(0) and Cu(ii), as reported by Hanf *et al.*,^31^ could contribute to the predominance of Cu(ii) and absence of a resolvable Cu(i) contribution in the QSCs. However, this interpretation remains tentative as the present measurements cannot distinguish unequivocally between Cu species associated with the current-collector interface and those present in the adjacent electrolyte.

### The graphite effect

The measurements conducted at OCP (pre-cycling) revealed a smaller oxide composition on the graphite-coated copper compared to the uncoated film as discussed above (see “Copper dissolution at OCP” and Fig. 1). Additionally, during *operando* measurements the graphite-coated copper showed the lowest overall percentage oxidation despite the relatively long cycling time. The effect of graphite in protecting copper was observed in previously reported cyclic voltammetry experiments where the graphite coating pushed the copper oxidation potentials to more positive values.^19^ These results indicate that copper oxidation occurs at a higher overpotential for graphite-coated copper in LIC compared to uncoated copper in AFC. This behaviour can be attributed to kinetic and interfacial effects introduced by the graphite layer. In particular, the coating limits direct electrolyte access to the copper surface and imposes a diffusion barrier for copper species generated at the interface, thereby suppressing dissolution. Consistent with the present observations, a separate study in which uncoated and graphite-coated copper foils were exposed to electrolyte for 14 weeks reported ∼50 ppm of dissolved Cu for the uncoated foil, compared with only trace levels (<1 ppm) for the graphite-coated foil.^36^ In addition, the formation of a passivating interphase on the graphite further reduces parasitic reactions. As a result, higher applied potentials are required to initiate significant copper oxidation. This agrees with study performed by Zhao *et al.*^19^ where authors used cyclic voltammetry scans to show that uncoated copper oxidised more readily with higher currents recorded, for the same potential range, compared to the graphite-coated one. The authors also showed increasing redox currents in successive cyclic voltammetry scans, which were attributed to the formation of additional layers of uncoated copper as dissolved ions re-plate on top of the graphite. The work here suggests that cycles of copper dissolution and re-deposition occur in lithium-ion batteries, and thus the continuous formation of uncoated copper on the graphite surface is also occurring.

### Copper Re-deposition on graphite and NMC

**Fig. 4:**
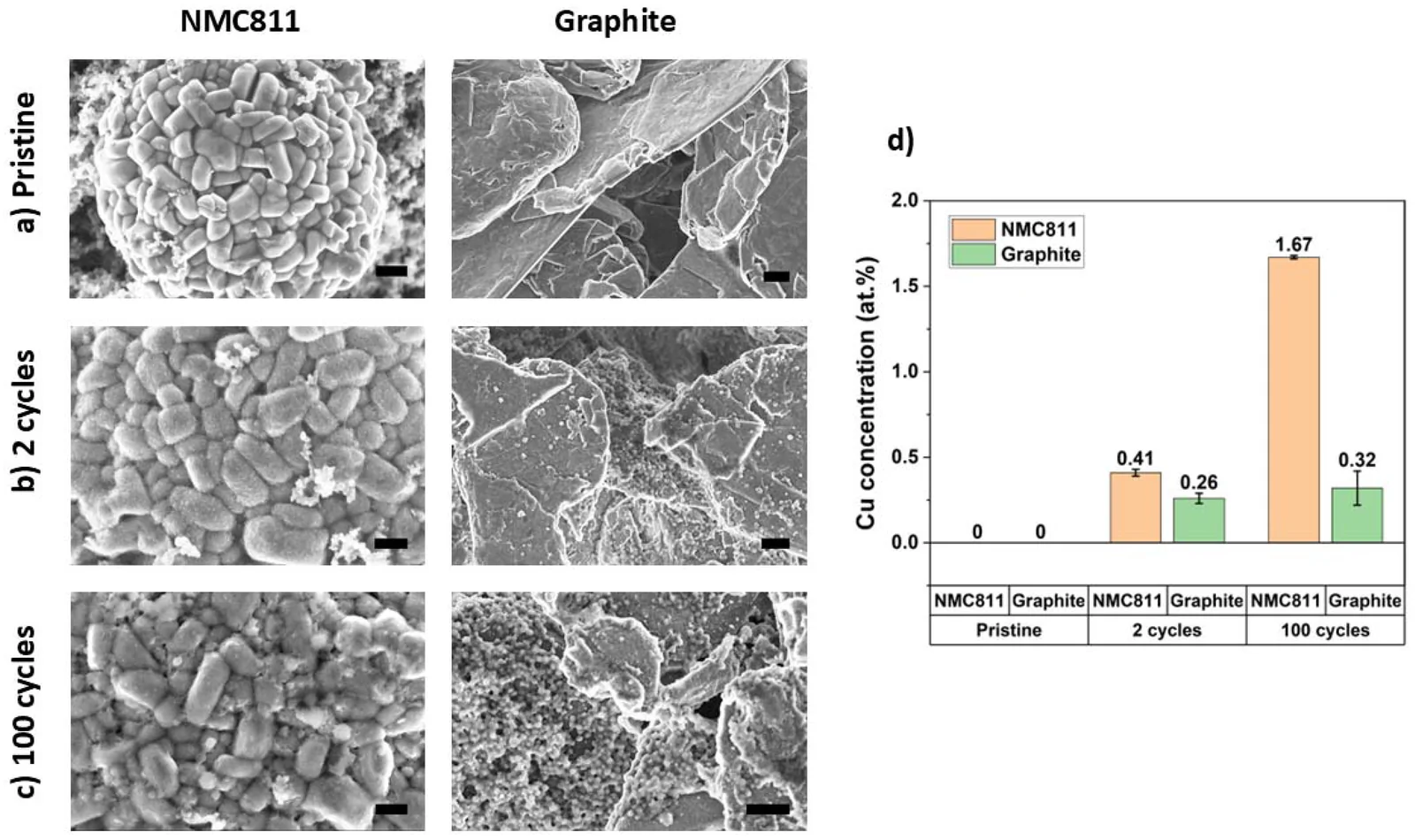
SEM-EDX of graphite and NMC electrodes revealing the concentration of copper (d) (at%) on (a) pristine electrodes, (b) after 2 cycles, and (c) after 100 cycles. Ten spectra were collected from each electrode and the mean copper concentration (at%) are reported plus/minus the standard deviation. Scale bars are 500 nm for NMC electrodes and 3 µm for graphite electrodes.

Energy dispersive X-ray spectroscopy in the scanning electron microscope (SEM-EDX) was used to detect copper deposition on graphite and NMC after cycling. Electrodes were extracted from LICs cycled at 2C for two cycles (similar to the LIC cell studied *in operando* with XAS) and 100 cycles. Copper deposits were detected on all cycled electrodes including those cycled for only two cycles. Ten EDX spectra were collected at various locations across each electrode, and the mean Cu atomic percentages are reported in Fig. 4. The low standard deviation values suggest a near uniform deposition of copper across the electrodes. Copper deposition was previously reported on anode, cathode and separator, but only in over-discharged cells,^14,21,22,24–27^ while the results here are evidence of copper dissolution within normal cycling potentials. Higher copper (at%) concentrations were detected on both electrodes after 100 cycles compared to 2 cycles suggesting that copper dissolution continues during cycling rather than only at OCP. This agrees with *in operando* observations where copper oxidation was detected during cycling.

### Impact of copper dissolution on battery safety, capacity and lifetime

#### Effect on battery safety

Copper dissolution poses safety risks primarily through the probability of dendrite formation and enhanced side reactions. Dissolved copper ions can re-deposit unevenly, forming metallic copper dendrites that may penetrate the separator,^37^ leading to internal short circuits and potential thermal runaway as has been previously observed in stress tested cells.^29^ This can be further exacerbated by fast charging. Furthermore, copper dissolution may catalyse electrolyte decomposition reactions, generating gases^14,29^ and increasing internal cell pressure, which elevates the risk of cell swelling, venting, or explosion. The parasitic redox reactions involving metallic copper on graphite also contribute to the instability of the electrode–electrolyte interface due to the enhanced surface area, potentially triggering uncontrolled lithium plating and dendrite growth, both critical safety hazards in lithium batteries.^25,29^

#### Effect on battery capacity

Copper dissolution in lithium-ion batteries leads to the formation of corrosion products that can increase the electronic resistance at the current collector–electrode interface. This increased resistance impedes efficient electron flow, thereby reducing the effective utilisation of active materials and decreasing the overall battery capacity. Additionally, dissolved copper ions can migrate and deposit on electrode surfaces, including the cathode and separator, potentially blocking lithium-ion transport pathways and active sites for lithium intercalation/de-intercalation.^24^ This surface blocking further limits the reversible capacity of the battery by hindering lithium-ion access to electrode materials. In a Cu-sputtering experiment used to simulate Cu dissolution, authors reported that the capacity of battery decreased with increasing amount of sputtered Cu.^26^

#### Effect on battery lifetime

The ongoing dissolution and re-deposition cycles of copper during normal battery operation contribute to progressive structural and interfacial degradation. Copper dissolution leads to thinning and fragmentation of the current collector, which can cause electronic disconnection of electrode regions, reduce the effective electrode area and accelerate capacity fade. The effect can be especially deleterious during pitting corrosion, which is autocatalytic, meaning that once established, conditions within the pit become more aggressive and promote localised dissolution. A study reported the occurrence of pits within normal cycling windows with more pits observed at higher C-rates.^38^ The deposition of copper on electrodes and separators introduces parasitic reactions that may destabilise the SEI^39^ and other protective layers, promoting further electrolyte decomposition and mechanical degradation. These cumulative effects shorten the battery's operational lifetime by exacerbating capacity loss and increasing internal resistance over repeated cycling.

#### Implications on battery cycling

Due to the pronounced effects of the copper dissolution on battery safety, capacity and lifetime, the practice of tap charging the battery is widely implemented in industrial and more recently in academic settings. The tap charging process in full cell lithium-ion batteries involves charging to and holding the cell potential at *ca.* 1.5 V after assembly to insert small amounts of lithium ions into the graphite anode. This process ensures that the potential of the anode remains below its corrosion threshold.^11,40^

The mechanistic insight of this study directly informs the operational rationale for tap charging practiced in the battery industry. Our XAS-resolved copper speciation data provide direct and quantitative evidence that copper current collector corrosion takes place in various lithium-ion cells both during rest at OCP and cycling within voltage windows that are conventionally thought to be away from the dissolution potentials. This is particularly relevant with the emergence of anode-less battery chemistries, where copper is directly exposed to the electrolyte without any graphite coating. Our data suggest that beyond the well-recognised dissolution pathways, additional corrosion mechanisms may become operative in such emerging chemistries. This highlights the need for more rigorous evaluation of copper stability in next-generation anode-less cells, which lack the buffering effects provided by conventional graphite anodes.

## Conclusions

This study demonstrates that copper dissolution and re-deposition on lithium-ion battery electrodes occurs even under normal cycling conditions. Crucially, this happens in all cell types studied here that include lithium-ion full cells (NMC811 *vs.* graphite), anode-free cells (NMC *vs.* Cu) and *quasi*-symmetrical cells (Cu *vs.* Li). This was previously unexpected given that copper is nominally stable in the perceived electrochemical conditions of normal battery operation. The oxidation and reduction of copper are parasitic reactions in lithium-ion batteries and raise concerns over their effect on the structure of the SEI and the efficiency of the lithium (de)intercalation processes. While these reactions had been previously predicted and observed only in over-discharged cells, the results here suggest that copper current collectors are implicit in limiting lithium-ion battery efficiencies and the implications of copper dissolution should be considered during normal cell operation.

## Author contributions

M. A. K. conceptualisation, data curation, formal analysis, investigation, methodology, validation, visualisation, writing original draft, writing – review & editing; A. O. formal analysis, visualisation, writing original draft, writing – review & editing; H. Y., W. X., J. S. Z., D. T., L. G. investigation, methodology; J. N. W. conceptualisation, methodology; J. F. W. M. resources, investigation, methodology, validation; M. K. methodology, validation, supervision; I. E. L. S. resources, methodology, writing – review & editing; M. P. R. conceptualisation, resources, methodology, supervision, writing – review & editing.

## Conflicts of interest

There are no conflicts to declare.

## Supplementary Material

EB-002-D6EB00072J-s001

## Data Availability

The data supporting this study are available in the article and its online supplementary information (SI). Supplementary information including examples of the linear combination fitting analysis carried out on XAS spectra and quality of fits (R-factor) analysis are available. See DOI: https://doi.org/10.1039/d6eb00072j.
