# Supplementary material for: Understanding the reactivity of copper current collectors in lithium batteries – an operando XAS study
Source: EES Batter. 2026 Aug 24;2(5):1668–77. doi: 10.1039/d6eb00072j (PMC13556142; doi:10.1039/d6eb00072j)
Supplement: Supplementary file 1 [file EB-002-D6EB00072J-s001.pdf]

Supplementary Information (SI) for EES Batteries.
This journal is © The Royal Society of Chemistry 2026

Electronic Supplementary Information for

"Understanding the Reactivity of Copper Current Collectors in Lithium Batteries – an *Operando* XAS Study"

M. A. Koronfel [a,b], A. Omirkhan [*a,b], H. Yadegari [a], D. Thornton [a,b], W. Xue [a], J. S. Zhang [a], L. Guo [a], J. Nelson-Weker[c], J. F. W. Mosselemans[d], M. Kulzick [e], I. E. L. Stephens [a,b], M. P. Ryan [*a,b]

a. Imperial College London, Department of Materials and London Centre for Nanotechnology, London, SW7 2AZ, UK

b. The Faraday Institution, Quad One, Harwell Science and Innovation Campus, Didcot, OX11 0RA, UK.

c. SLAC National Accelerator Laboratory, Menlo Park, 94025-7015, USA

d. Diamond Light Source, Rutherford Appleton Laboratory, Didcot, OX11 0DE, UK

e. BP Corporate Research Center, Naperville, Illinois, 60563, USA

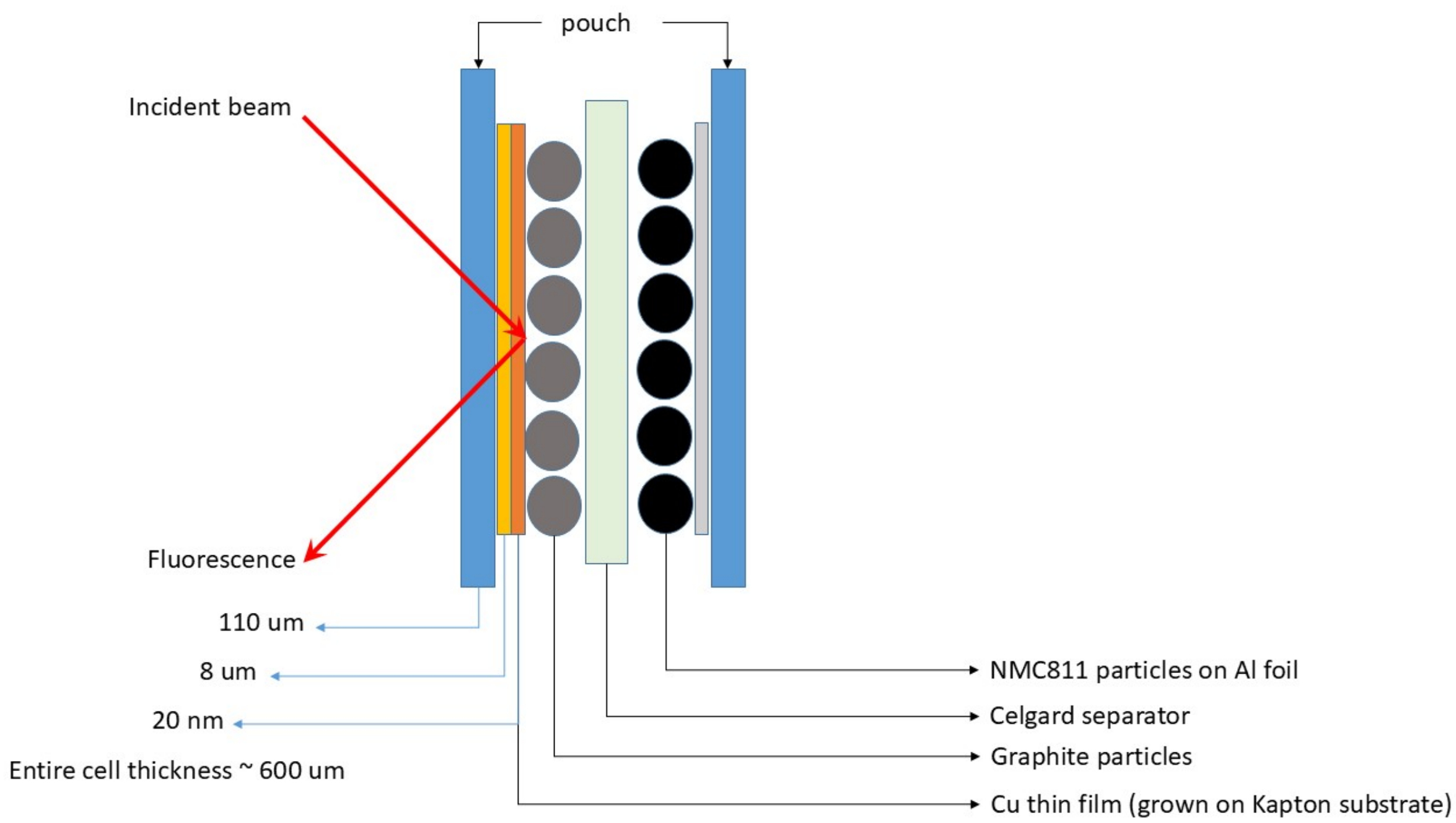

ESI Figure 1. The schematic of X-ray transparent battery pouch cells used in the study

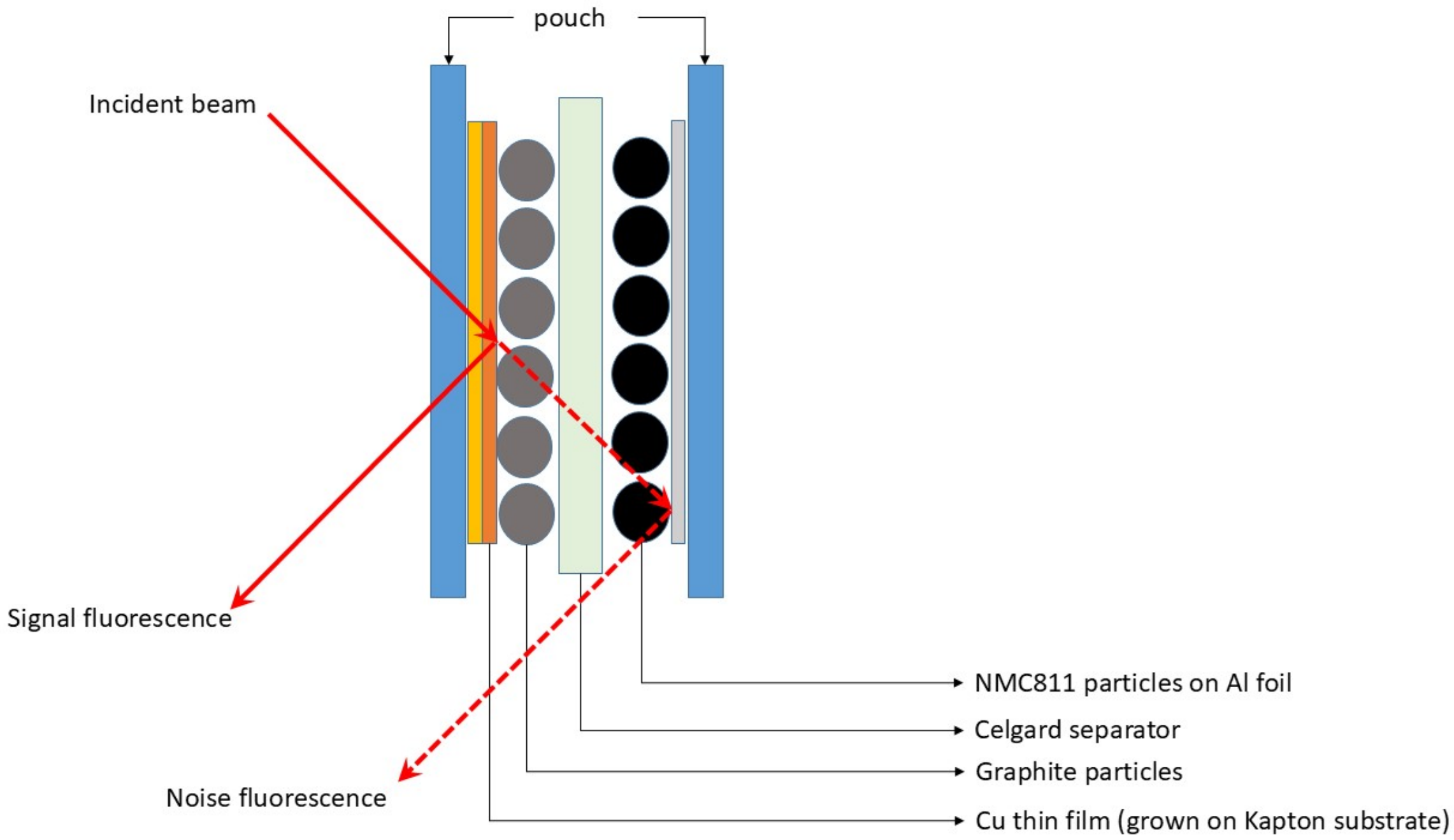

ESI Figure 2. Fluorescence geometry showing the orientation of the cell at 45^0 and the detector at 90^0 to the incident beam.

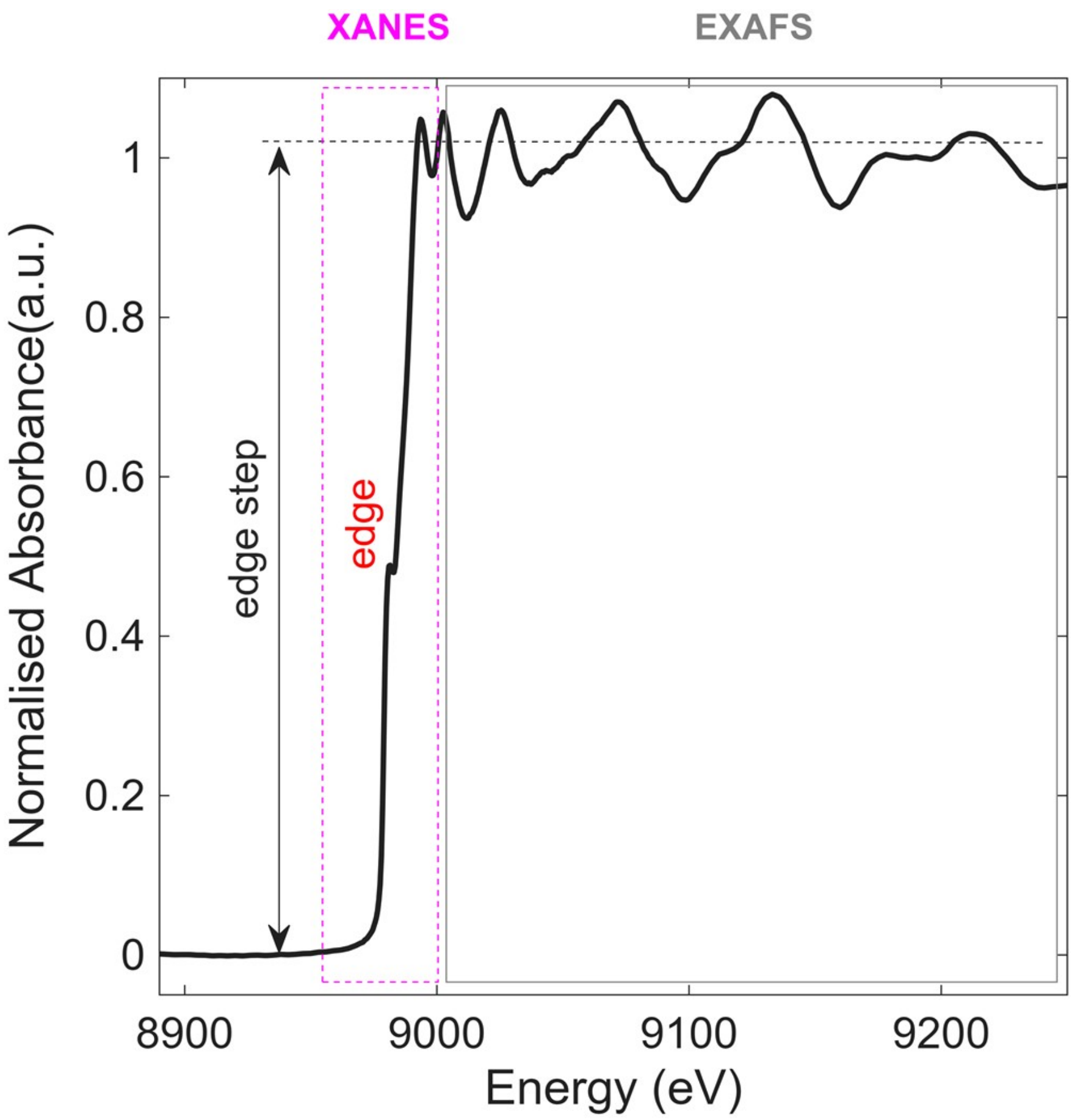

ESI Figure 3. Representative graph showing normalised X-ray Absorption data with X-ray absorption near edge spectroscopy (XANES) and extended X-ray absorption fine structure (EXAFS) regions.

Each element has a characteristic set of absorption edges corresponding to the energy of its electrons. Absorption energy increases with the atomic number of the element. Scanning the X-ray energy across a particular edge (*e.g.* Cu K-edge) results in a distinctive X-ray absorption spectrum. The region of the spectrum near the absorption **edge** is XANES, while the region 50-100 eV beyond the edge is called EXAFS.

The oxidation of the element (*e.g.* Cu to $Cu^{+/++}$) shifts the absorption edge to a higher X-Ray energy. Change in the oxidation state of the element will also be accompanied by the changes in the features at the absorption edge. Importantly, in XAS the edge-step height is directly proportional to the concentration of the absorbing element as per Beer-Lambert Law (ESI Equation 1). For samples containing two or more oxidation states of the element, linear combination fitting (LCF) can be used to determine the composition of each species.

$$I = I_0 e^{-\mu x}$$ ESI Equation 1,

Where I is the intensity of the transmitted beam, I_0 is the intensity of the incident X-Ray beam, x is the thickness of a sample and μ is the linear attenuation coefficient describing the fraction that gets absorbed or scattered per unit thickness of a sample. μx is defined as absorbance.

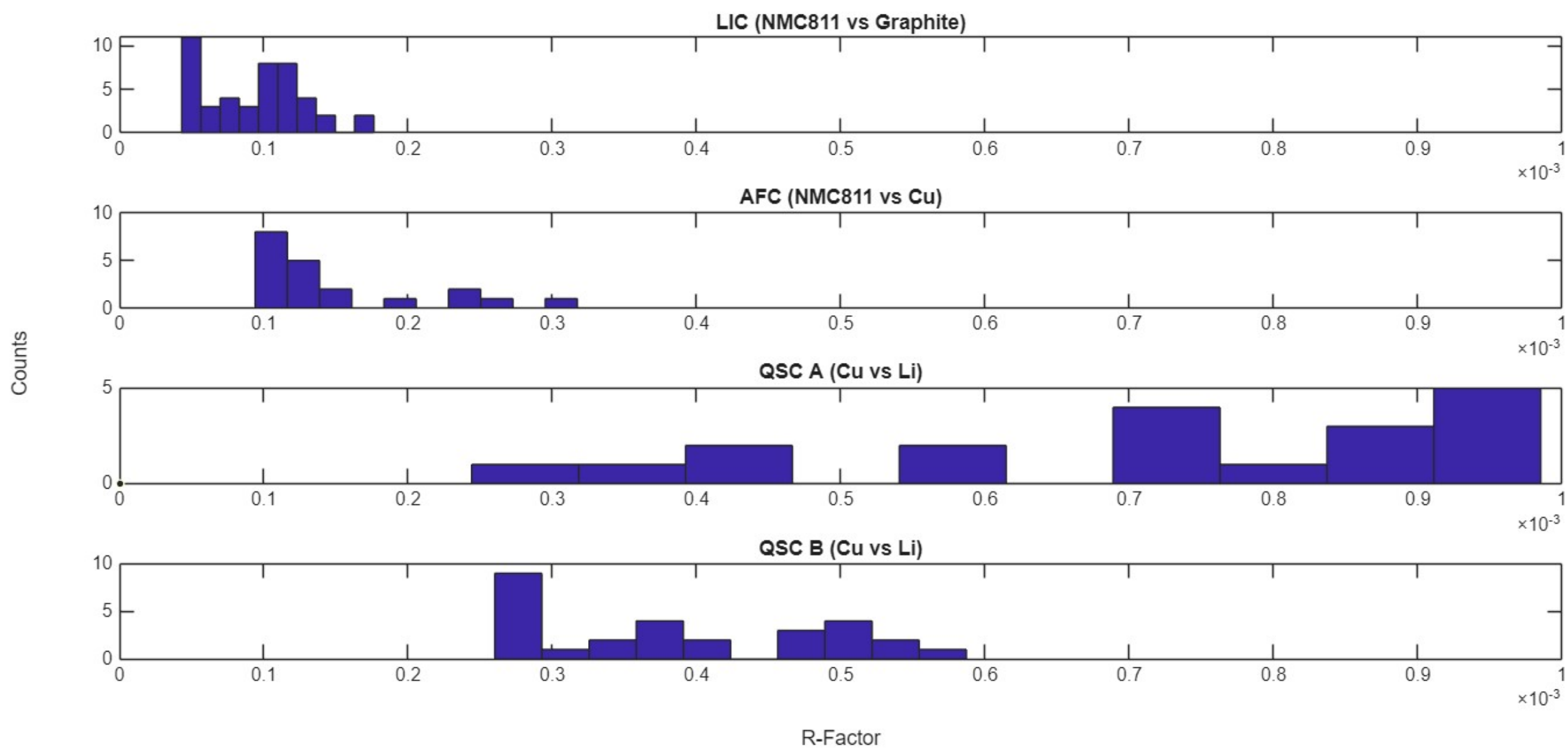

ESI Figure 4. Distribution of R-factor values obtained from XAS spectral fitting for four electrochemical systems: LIC (NMC811 *vs* graphite), AFC (NMC811 *vs* Cu), and QSC configurations (Cu vs Li, A and B). The R-factor provides a quantitative measure of the agreement between experimental and fitted spectra, with lower values indicating better fit quality. The LIC system exhibits consistently low and narrowly distributed R-factors, reflecting robust fits, while the broader and higher distributions observed for the anode-free and QSC systems point to increasing chemical and interfacial complexity, particularly pronounced in QSC A.

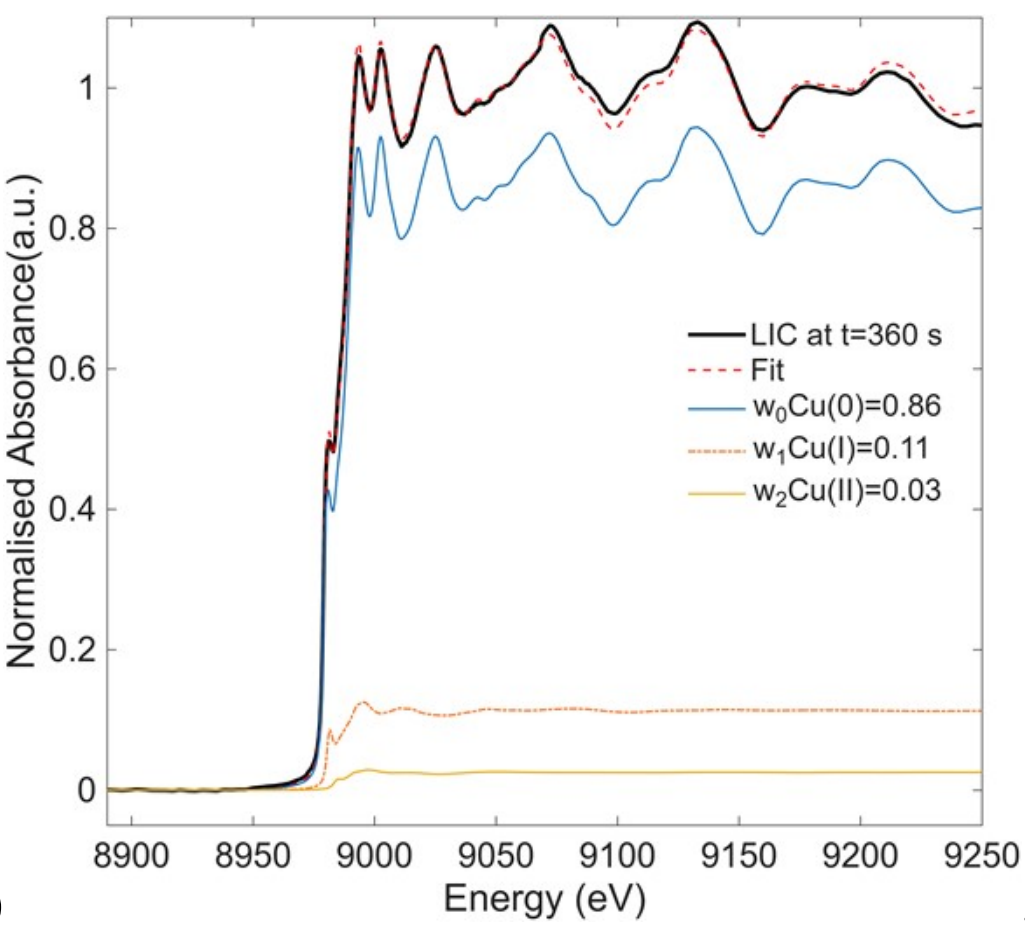

a)

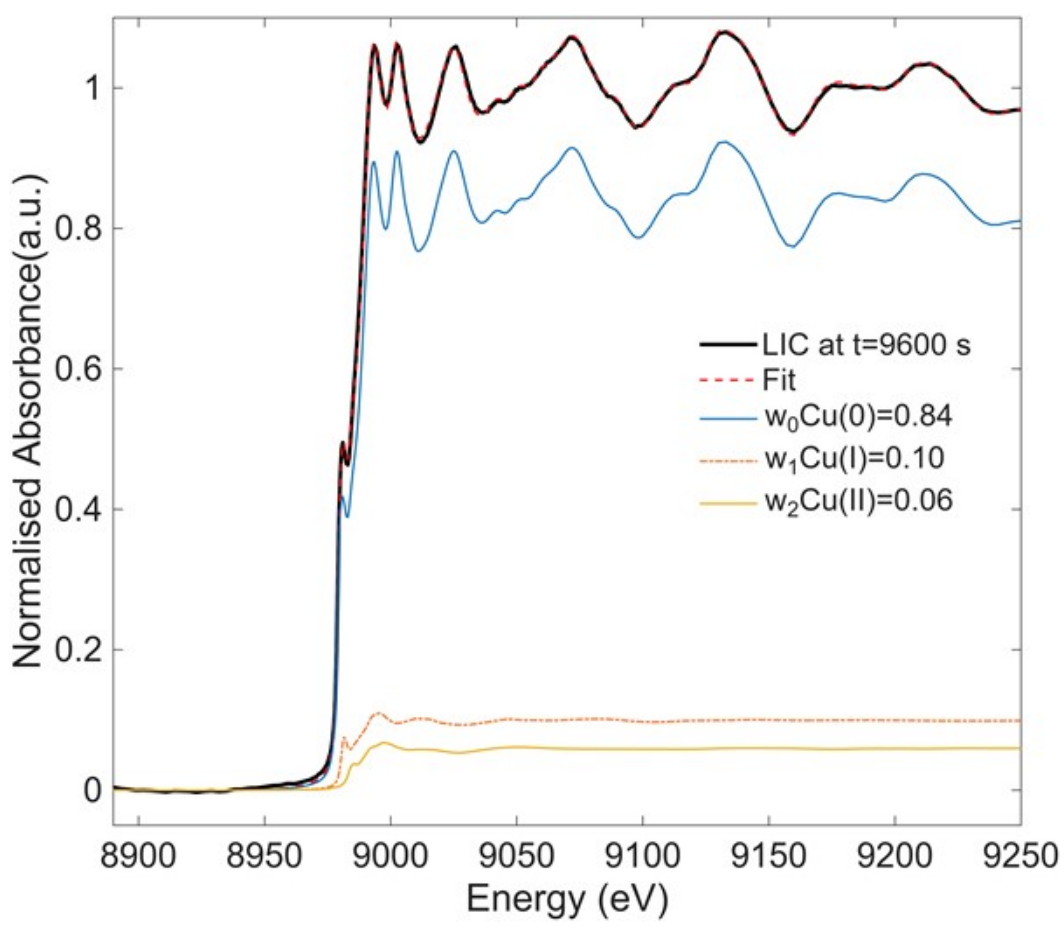

, b)

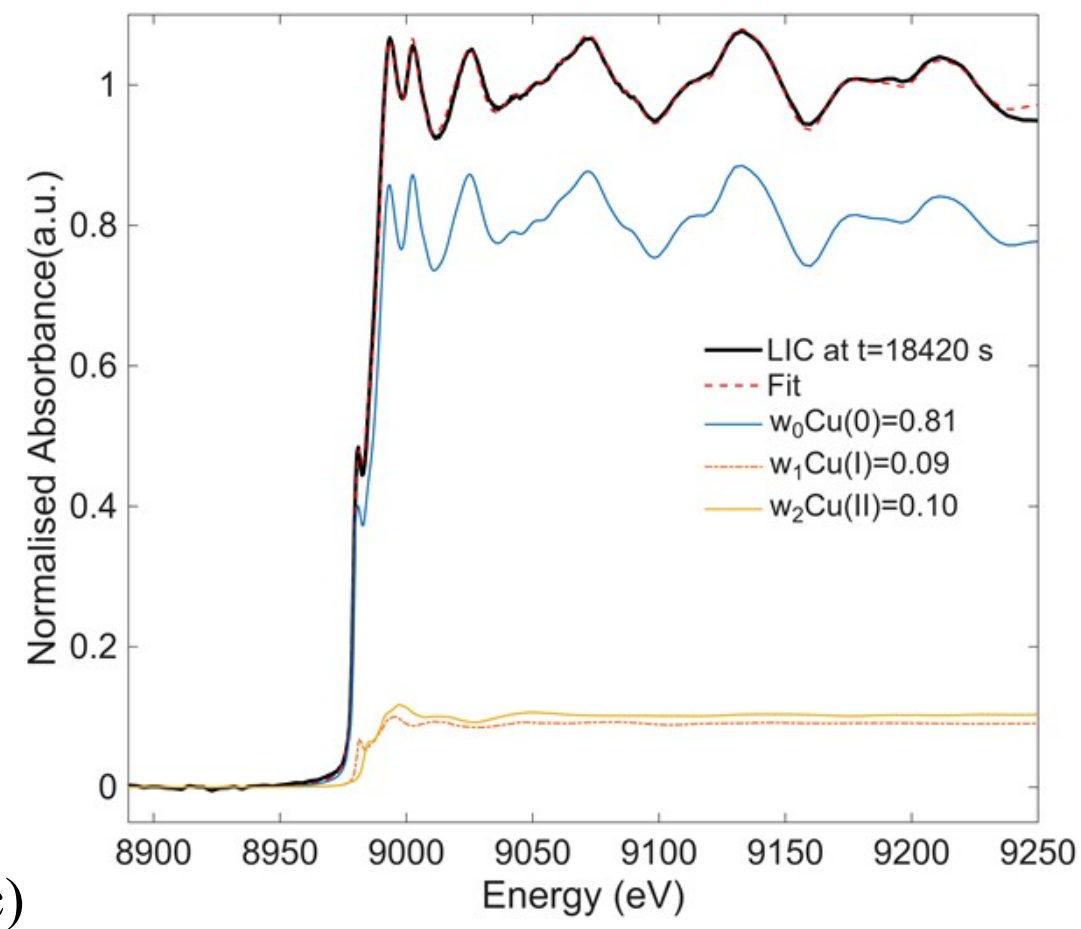

c)

ESI Figure 5. LCF results of graphite coated copper films in LIC revealing their chemical composition at a) t=360s, b) t=9600s, c) t=18420s during electrochemical cycling.
